# Production of Bioactive Peptides by *Lactobacillus* Species: From Gene to Application

**DOI:** 10.3389/fmicb.2018.02354

**Published:** 2018-10-17

**Authors:** Cyril Raveschot, Benoit Cudennec, François Coutte, Christophe Flahaut, Marc Fremont, Djamel Drider, Pascal Dhulster

**Affiliations:** ^1^INRA, ISA, EA 7394-ICV Institut Charles Viollette, Université Lille, Université d’Artois, Université Littoral Côte d’Opale, Lille, France; ^2^VF Bioscience, Parc Eurasanté, Loos-lez-Lille, France

**Keywords:** bioactive peptides, fermentation, hydrolysis, *Lactobacillus*, strain screening

## Abstract

To compensate for their amino acid auxotrophy, lactobacilli have developed the ability to hydrolyze proteins present in their environment. This proteolytic activity not only generates the free amino acids needed by the bacteria, but also a large variety of peptides, some of which are endowed with biological activities. These so-called “bioactive peptides” (BAPs) are interesting from a nutrition and healthcare perspective. The use of lactic acid bacteria (LAB) such as lactobacilli is an effective strategy for production and valorization of new BAPs. The proteolytic activity of lactobacilli is exerted in a strain- and species-dependent manner: each species exhibits different proteinase content, leading to a large variety of proteolytic activities. This underlines the high potential of *Lactobacillus* strains to produce novel hydrolysates and BAPs of major interest. This review aims at discussing the potential of different *Lactobacillus* species to release BAPs from fermentation media and processes. Strategies used for peptide production are presented. Additionally, we propose a methodology to select the most promising *Lactobacillus* strains as sources of BAPs. This methodology combines conventional approaches and *in silico* analyses.

## Introduction

It is now recognized that diet plays a key role in the maintenance of our health status. More and more research projects aim at determining how diet could improve or restore health (Cornier et al., 2008) and a new discipline, Foodomics, was defined in 2009 as “a discipline that studies the Food and Nutrition domains through the application of advanced Omics technologies to improve consumer’s well-being, health and confidence” (Cifuentes, 2009; Braconi et al., 2017). Foodomics could, for instance, help identify approaches to reduce the prevalence of metabolic syndrome (O’Neill and O’Driscoll, 2015; Chen et al., 2018), which is defined as a cluster of symptoms (high blood pressure, high blood sugar, overweight, hyperlipidemia) ultimately increasing the risk of diabetes, stroke, cardiovascular diseases, or cancers (Cameron et al., 2004; Wilson et al., 2005; Cornier et al., 2008; Kassi et al., 2011).

Food proteins are currently being studied beyond their nutritional characteristics, for their positive impact on human health related to specific sequences encrypted into the native protein, called bioactive peptides (BAPs). Such sequences are inactive when present in the parental protein, but can be released after protein hydrolysis during gastro-intestinal digestion (GID), *in vitro* enzymatic hydrolysis, or microbial fermentation (Korhonen and Pihlanto, 2006; Korhonen, 2009). The BAPs display interesting biological functions such as angiotensin converting enzyme (ACE) inhibition, mineral binding, antidiabetic, satiating, immunomodulating, opioid, antioxidant, or antimicrobial activities (Nongonierma and FitzGerald, 2015; Park and Nam, 2015; Caron et al., 2017; Nielsen et al., 2017; Domenger et al., 2018). Research on BAPs provides insightful information on the impact of dietary proteins on health. Furthermore, industrial applications are emerging such as the production of functional foods (Korhonen and Pihlanto, 2006; Pihlanto, 2013; Hafeez et al., 2014) or the production of peptides to serve as active ingredients for pharmaceuticals or dietary supplement products.

Challenges for production and commercialization of BAPs include: (i) the identification and isolation of new BAPs, (ii) the elucidation of the mechanisms of action involved in their bioactivities, and (iii) the development of efficient bioprocesses for peptide production (Agyei et al., 2016; Giacometti and Buretić-Tomljanović, 2017; Nongonierma and FitzGerald, 2017b). Research on BAPs is progressing. The use of *in silico* methodologies facilitates the discovery and identification of new peptides (Sánchez-Rivera et al., 2014; Iwaniak et al., 2015). However, the industrial production of BAPs is still limited by the lack of suitable large-scale technologies and the high cost of enzymes used for protein hydrolysis (Wu et al., 2013).

Microbial fermentation represents a cost-effective method for BAP production (Daliri et al., 2017), already widely applied in the dairy industry for the functionalization of milk products and byproducts (Pihlanto, 2013; Hafeez et al., 2014). Fermented dairy products claiming functional health properties associated with BAPs already exist. Most of these products are obtained using LABs belonging to the *Lactobacillus* genus (Korhonen and Pihlanto, 2006; Hafeez et al., 2014).

The LAB constitute a diverse group of Gram-positive, catalase-negative bacteria producing lactic acid as the main end-product of carbohydrate fermentation (Felis and Dellaglio, 2007). With more than 231 valid species and 29 subspecies, *Lactobacillus* genus is certainly the main and most diverse LAB group. Lactobacilli are bacteria of major industrial interest, as it is used for the production of fermented foods (dairy, meat, or vegetable products), food biopreservation, or probiotic applications (Stefanovic et al., 2017). However, they are considered as fastidious microorganisms because of their auxotrophy for numerous amino acids. In order to find the amino acids required for their growth, lactobacilli hydrolyze proteins in their environment through their proteolytic system, and, more specifically, through the action of enzymes called cell envelope proteinases (CEPs). This leads to the release of peptides and free amino acids in the fermentation media (Savijoki et al., 2006). Besides contributing to changes in the organoleptic properties of the fermented product, these released peptides can display various biological activities (Hafeez et al., 2014). Numerous studies reported the presence of BAPs in ripened cheeses (Gouda, Cheddar, Swiss cheese varieties, Manchego) (Pihlanto, 2013) or in yogurts (Jin et al., 2016; Rutella et al., 2016). The most meaningful examples of BAPs produced by *Lactobacillus* species are the antihypertensive tripeptides, Ile-Pro-Pro and Val-Pro-Pro (also called lactotripeptides), generated from casein hydrolysis by different *Lb. helveticus* strains (Pihlanto, 2013). Fermented milk products containing antihypertensive BAPs produced by *Lactobacillus* strains are already being marketed (Korhonen and Pihlanto, 2006; Hafeez et al., 2014).

Lactobacilli are isolated from different ecological niches. Various studies have described large differences between species or even strains, at the genetic or physiological levels (Hutkins, 2006; Sun et al., 2015); it is established that different strains belonging to the same species can differently hydrolyze proteins (Jensen et al., 2009). This not only supports the idea that lactobacilli can produce a large variety of BAPs, but also highlights the need to carefully select the strains that will be used for BAP production.

This review describes the proteolytic system of *Lactobacillus* species, focusing on the aptitude of these microorganisms to hydrolyze dietary proteins in order to produce novel BAPs. A method for selection of BAP-producing *Lactobacillus* strains is proposed, based on a combination of conventional and *in silico* approaches. The strategies for BAP production using *Lactobacillus* species are presented, including (i) fermentation and functionalization of dietary products, (ii) extraction and purification of hydrolysates or BAPs, and (iii) use of purified CEPs. The advantages and drawbacks of each strategy are discussed. Finally, the current limitations and future challenges for BAP production by lactobacilli are discussed.

## The Proteolytic System of *Lactobacillus* Species and Its Potential to Produce a Broad Variety of Peptides

**FIGURE 1:**
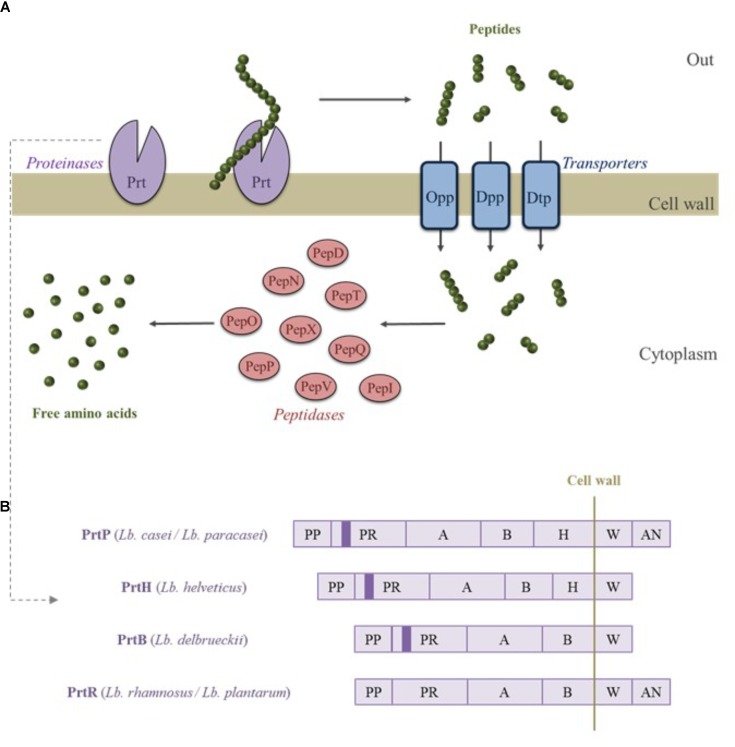
Schematic representation of the proteolytic system of *Lactobacillus* spp. **(A)**. Protein hydrolysis is initiated by cell envelope proteinases (CEPs and Prt) and the resulting peptides are then transported inside the cell. Peptides are finally transformed into free amino acids by different peptidases. Diagrammatic representation of CEPs from different *Lactobacillus* species **(B)**. PP, pro domain; PR, catalytic domain; A, A-domain; B, B-domain; H, helical domain; W, cell wall spacer domain; AN, membrane anchor domain. The plain color in the catalytic domain represent the insertion I domain. Adapted from Sadat-Mekmene et al. (2011a).

*Lactobacillus* species are auxotrophic for numerous amino acids. An external source of nitrogen is required for their growth, particularly in milk where concentrations of amino acids are low. To fulfill their nitrogen requirement, *Lactobacillus* species have developed a proteolytic system, which hydrolyzes the proteins and supplies the amino acids. This system is composed of three major components (**Figure 1A**). Protein hydrolysis is initiated by CEPs that cleave the proteins into peptides ranging from 4 to 30 amino acids. As demonstrated by gene deletion experiments, CEPs are required for the strain’s growth in milk (Courtin et al., 2002). Peptides generated by CEP cleavage, released in the extracellular media, are then internalized by different transporters such as the oligopeptide permease (Opp), the ion-linked transporter (DtpT) for di- and tripeptides, and the ABC transporter (Dpp) for peptides containing 2 to 9 amino acid residues. Subsequently, the internalized peptides are degraded into amino acids by the combined action of numerous internal peptidases such as endopeptidases (PepO, PepF, PepE, and PepG), aminopeptidases (PepN, PepC, PepS, PepA, and PepL), tripeptidases (PepT), dipeptidases (PepD and PepV), and proline-specific peptidases (PepQ, PepI, PepR, PepX, and PepP) (for reviews see Savijoki et al., 2006; Sadat-Mekmene et al., 2011a; Griffiths and Tellez, 2013).

The CEPs are serine proteinases that belong to the subtilisin family. These enzymes are synthesized as preproteins of 2,000 amino acids and composed of 7 functional domains (**Figure 1B**). From the N terminus side, CEPs include the protein pro domain (PP), the catalytic domain (PR), an insertion domain (I) that possibly modulates substrate specificity, the domain A (unknown function), the domain B probably involved in CEP stabilization, the helix domain (H) that keeps the CEPs outside the cell, and a hydrophilic domain (W) (cell wall spacer or cell wall binding domain) (Savijoki et al., 2006). An anchor domain (AN) is present at the C-terminus of the protein except for the CEPs of *Lb. helveticus* and *Lb. delbrueckii* subsp. *bulgaricus* species (Griffiths and Tellez, 2013).

Four different CEPs from *Lactobacillus* species have so far been characterized. They include PrtB from *Lb. delbrueckii* subsp. *bulgaricus* (Hou et al., 2015), PrtP from *Lb. casei* and *Lb. paracasei* (Kojic et al., 1991; Ikram-ul-Haq and Mukhtar, 2006; Alcántara et al., 2016), PrtR from *Lb. rhamnosus* and *Lb. plantarum* (Vukotić, 2016), and PrtH from *Lb. helveticus* (Pederson et al., 1999; Griffiths and Tellez, 2013). While most *Lactobacillus* species possess only one CEP, the presence of four different paralogs of PrtH (called PrtH1, PrtH2, PrtH3, and PrtH4) has been observed in *Lb. helveticus* and their distribution is strain-dependent (Broadbent et al., 2011; Miyamoto et al., 2015). The presence of several CEP paralogs, exhibiting different specificities, make *Lb. helveticus* the most proteolytic species among the *Lactobacillus* genus and obviously the most efficient at generating a great diversity of BAPs (Genay et al., 2009; Stefanovic et al., 2017). Moreover, the proteolytic system of *Lb. helveticus* is the most studied due to its wide utilization for the production of dairy products, mainly cheese and fermented milk (Broadbent et al., 2011).

Proteolytic activities and protein hydrolysis patterns differ widely from one strain to another (Jensen et al., 2009). This is probably due to multiple factors like differences in CEP gene expression, CEP gene mutations, differences in optimal conditions for enzymatic activity, although the methodology used to assess the hydrolysis pattern of CEPs may also somewhat introduce variability in the reported results (Sadat-Mekmene et al., 2011a; Patel and Hati, 2018). In the case of *Lb. helveticus* strains, the genetic diversity of CEPs explains at least in part the differences in protein hydrolysis patterns (Sadat-Mekmene et al., 2011b). Broadbent et al. (2011) reported that among 51 strains of *Lb. helveticus* of dairy origin, 6, 4, 21, and 20 of them possessed 4, 3, 2, or 1 CEP paralogs, respectively. The predominant paralog was PrtH3. Miyamoto et al. (2015) studied 6 strains isolated in Mongolia and reported different protein hydrolysis patterns than in strains isolated in European or North American countries. The only paralog detected in the 6 Mongolian strains was PrtH1. Hydrolysis pattern differences were also observed between different *Lb. delbrueckii* subsp. *bulgaricus* strains (Oberg et al., 2002; Oommen et al., 2002), although this species is known to possess only one CEP (PrtB).

As expected, the differences in proteolytic activities and protein hydrolysis patterns are even more visible when comparing different *Lactobacillus* species. The caseinolytic specificities of *Lb. helveticus* strains and *Lb. delbrueckii* subsp. *lactis* CRL581 strain are different (Hebert et al., 2008). A similar observation was made when comparing 14 strains of *Lb. delbrueckii* subsp. *bulgaricus* with 8 strains of *Lb. helveticus* (Oberg et al., 2002).

All these results show that *Lactobacillus* strains have different hydrolytic specificities for proteins and consequently may release very different BAPs. Some investigations are still needed to fully appreciate the diversity in CEP activities. A recent comparative genomic study of 213 *Lactobacillus* genomes allowed the detection of 60 different genes presenting significant homology with the currently known CEP genes (amino acid identities ranging from 100 to 20%). This high level of divergence shows the potential of *Lactobacillus* species to generate a large variety of different peptides (Sun et al., 2015).

Another source of BAP diversity is the type of protein used as substrate by the bacteria. Lactobacilli can indeed hydrolyze different types of proteins and BAP release could be tightly matrix dependent (Ayyash et al., 2017). Overall, *Lactobacillus* strains are mostly used for milk fermentation and most of the BAPs characterized so far were isolated from milk cultures (Dziuba and Dziuba, 2014). However, even milk proteins are not identical, and the same *Lactobacillus* strain will generate different peptides when hydrolysing caseins from cow milk, goat milk, camel milk, or mare milk. Moreover, *Lactobacillus* strains are used to hydrolyze proteins from other sources than milk, such as fish (Yang et al., 2016) or plants (Rizzello et al., 2017; Singh and Vij, 2017), and this can also lead to the release of specific BAPs. For example, fermentation of pea seed by *Lb. plantarum* 299v leads to the release of ACE inhibitor peptides (Jakubczyk et al., 2013).

In conclusion, *Lactobacillus* species clearly present a great potential as producers of new BAPs, due to (i) the presence of different CEPs, with species- and strain-specific activities, able to generate a large variety of peptides of different molecular weights and sequences and (ii) their capacity to hydrolyze different types of proteins. Studying CEP activities and specificities will lead to a better understanding of the health potential of dietary proteins.

## Selection of BAP-Producing *Lactobacillus* Strains

The basic strategy for the production and the characterization of new BAPs consists in the selection of a substrate and a proteolytic enzyme or microbial/bacterial strain, followed by hydrolysis under specific conditions (Korhonen, 2009). The resulting hydrolysate is assessed *in vitro* for biological activity and identification of BAPs. However, this strategy presents some limitations such as the empirical selection of substrate and enzyme as well as a lack of standardized protocols for the *in vitro* assessment of the biological activities. Recently, the development of dedicated software and computational tools has led to the development of more targeted/specific strategies, offering cost-effective and time-saving alternatives to conventional approaches (Sánchez-Rivera et al., 2014; Nongonierma and FitzGerald, 2017b). These approaches seem reasonably accurate, at least when predicting the outcome of hydrolysis by purified enzymes (Iwaniak et al., 2015). For example, PeptideCutter software (Gasteiger et al., 2005) is used to mimic protein/peptide hydrolysis and predict the sequence of the released peptides. Such predictions can help selecting the appropriate enzyme or protein source to generate specific BAPs (Carrasco-Castilla et al., 2012).

**FIGURE 2:**
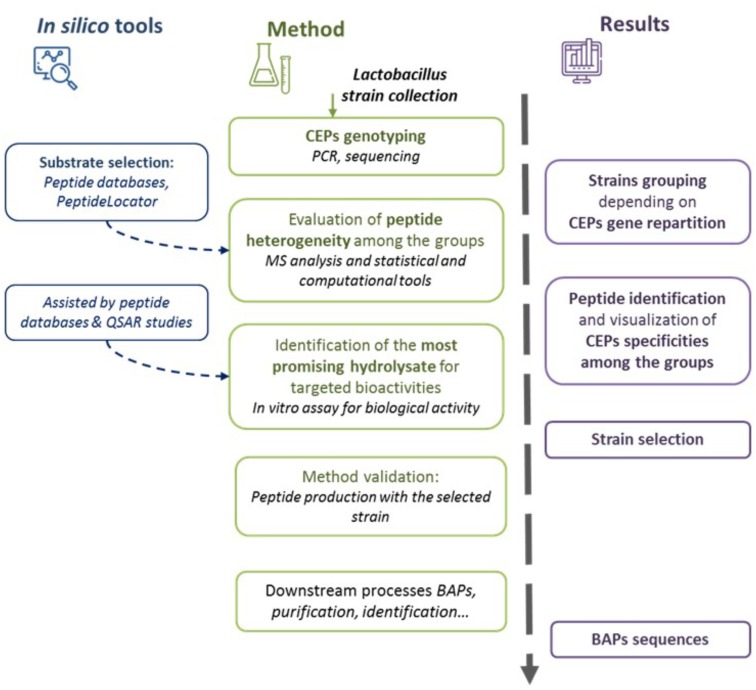
Schematic representation of the proposed methodology of *Lactobacillus* strains selection to produce bioactive peptides (BAPs). Cell envelope proteinase (CEPs) genotyping is performed, followed by the evaluation of the heterogeneity of the produced peptides. The most promising hydrolysate is selected depending on the targeted application. Finally, the selected strains are tested for method validation. Different *in silico* tools could be used during the methodology.

Regarding the production of BAPs using *Lactobacillus* species, the inter- and intra-genus variability of proteolytic activity and specificity make strain selection difficult. A methodology for selection of BAP-producing *Lactobacillus* strains is proposed (**Figure 2**). This selection procedure combines the use of a conventional approach with targeted strategies based on *in silico* analysis that were so far mostly used for BAPs produced by purified enzymes or gastro-intestinal enzymes (Nongonierma and FitzGerald, 2017a).

First of all, it is important to evaluate the inter- or intraspecific diversity of CEPs in a given *Lactobacillus* collection. The repartitioning of CEPs will indeed impact the proteolytic pattern diversities (Sadat-Mekmene et al., 2011b). This is particularly the case for *Lb. helveticus* isolates in which four different CEP paralogs can be present (Broadbent et al., 2011; Miyamoto et al., 2015). A rapid way to assess this diversity is the detection of CEP-encoding genes by PCR (Genay et al., 2009), by multiplex PCR (Broadbent et al., 2011), or even by genomic sequencing (Sun et al., 2015). It must be noted that when seven different CEPs have so far been characterized for *Lactobacillus* species (PrtB, PrtP, PrtR, and 4 paralogs of PrtH), it cannot be excluded that other, not yet described variants do exist in certain species (as noted earlier, searches based on gene homology have already identified 60 CEP gene candidates). Further work must be undertaken to characterize new CEPs from different species. In any case, strains presenting the same CEP genotype can be grouped together as likely to generate fairly similar proteolytic patterns.

The protein source must then be selected. A number of software can be useful at this stage. PeptideCutter is not relevant for use in the context of *Lactobacillus* research because CEPs are not yet covered by this software, probably due to a lack of sufficient enzymatic characterization and difficulties to model CEPs specificities. Protein selection should be done using BAP databases to search for bioactive sequences in different proteins of interest. Numerous BAP databases exist, usually more or less related to specific activities or sources (for review see Iwaniak et al., 2015; Blanco-Míguez et al., 2017; Giacometti and Buretić-Tomljanović, 2017; especially for milk proteins see Nielsen et al., 2017). The PeptideLocator software (Mooney et al., 2013) is dedicated to the prediction of BAPs in protein sequences. The ToxinPred software could also be used for predicting toxic peptide sequences and avoid selection of a highly cytotoxic protein (Gupta et al., 2013).

Following incubation of the selected protein with different CEP-harboring *Lactobacillus* strains (or extracted CEPs), the released peptides can be identified using mass spectrometry. This peptidomics analysis allows to assess the proteolytic potential of the selected strains or their CEPs (Hernández-Ledesma et al., 2004; Dallas et al., 2016; Giacometti and Buretić-Tomljanović, 2017). A peptide coverage diagram can be constructed, which highlights the protein areas that are preferentially hydrolyzed (Dallas et al., 2015). The Peptigram software could be used for peptidomics data visualization (Manguy et al., 2017). Moreover, different computational representations or statistical analyses can be used to assess peptide distribution among different samples or conditions as the Venn diagram, the principal component analysis (PCA), multiple linear regression (MLR), artificial neural networks (ANNs), or partial least square-discriminant analysis (PLS-DA) (Iwaniak et al., 2015; Dallas et al., 2016; Jin et al., 2016; Li et al., 2017). Therefore, peptidomics will be able to establish correlations between the CEP genotype of the strains and the profile of peptides they generate.

The selection of *Lactobacillus* strains is based on the bioactive potential of the produced peptides. Bioinformatics tools can be used to predict the biological activities of peptides. Through BAP databases, bioactive sequences can be easily identified by homology search against known functional peptide sequences (Iwaniak et al., 2015; Blanco-Míguez et al., 2017; Giacometti and Buretić-Tomljanović, 2017; Nielsen et al., 2017). The PeptideRanker server was designed for the prediction of BAPs activities (Mooney et al., 2012). Numerous studies have used quantitative structure activity relationship (QSAR) modeling to achieve peptide activity prediction (Carrasco-Castilla et al., 2012). The QSAR models analyze the correlations between the structural characteristics of molecules and their biological activities in order to predict (even quantitatively) the bioactivity of different peptides. The development of a QSAR model (for review see Iwaniak et al., 2015; Nongonierma and Fitzgerald, 2016a) is first conducted by the construction of a BAPs library composed of known functional sequences with quantitative biological data (such as half maximal inhibitory/effective concentration, IC_50_/EC_50_). The BAPs are then described by the creation of different descriptors such as physicochemical properties or amino acid scalar descriptors. Once all the BAPs from the library are described, the QSAR is mathematically modeled using computational methods and statistical analysis (for example PCA, MLR, ANNs, or PLS). Finally, the QSAR model is cross-validated by a test set of BAPs and by confirmatory studies with synthetic peptides. Different QSAR models have not only been developed mainly for ACE inhibition activity (for review see Jahangiri et al., 2014), but also for dipeptidyl dipeptidase IV (DPP-IV) activity (Nongonierma and Fitzgerald, 2016b) and α-glucosidase inhibition (Mollica et al., 2018). The use of QSAR modeling is an interesting approach. Although there is a risk of missing novel peptides with unknown bioactive properties since the method only takes into consideration the sequences that already exist in peptide databases. The main limitation of QSAR is related to the BAP dataset construction. BAPs should preferentially be identified and characterized by the same *in vitro* methods (Nongonierma and Fitzgerald, 2016a). Furthermore, there is also a need to develop more sophisticated amino acid and peptide descriptors as well as to improve the mathematical expression and algorithms for QSAR modeling (Agyei et al., 2016).

Bioinformatics tools can, therefore, be very useful to select the most promising strains, protein sources, and even predict the activities of the generated peptides. However, it is obvious that an *in vitro* assessment of the predicted BAP activity must be ultimately undertaken to validate *in silico* predictions (Lafarga et al., 2015).

## Methods for BAPs Production by *Lactobacillus* Species

Different strategies have already been described for the production of BAPs by *Lactobacillus* species or their CEPs. These include functionalization of food products by fermentation; the purification of peptides from fermentation broth; and finally, the use of purified or partially purified *Lactobacillus* CEPs.

In this section, these strategies are presented and updated based on recent technological progress. Each strategy has different advantages and drawbacks, which are presented in **Table 1**. The choice will mostly depend on the targeted application. Different applications of these methodologies are reviewed in **Table 2**.

**Table 1:** Methodologies for BAP production using lactobacilli: advantages and disadvantages.

Strategy for BAP production by lactobacilli	Advantages	Disadvantages	Reference
Fermentation of food products	Development of functionalized food products	Biological effect could be attributed to peptides released by gastro-intestinal digestion of non-hydrolyzed proteins	Wakai and Yanamoto, 2012; Hafeez et al., 2014; Daliri et al., 2017; Li et al., 2017; Marco et al., 2017
	Simultaneous production and functionalization	Biological effect could be attributed to other compounds produced during fermentation	
	No need for BAP extraction or purification		
BAP extraction/purification from fermented broth	Many applications (food, pharmaceuticals, and cosmetic)	Downstream processes of extraction or purification are expensive	Korhonen and Pihlanto, 2006; Agyei et al., 2016


	No interferences with other compounds produced during fermentation		
	Could lead to BAP enrichment in the final product		
Utilization of partially purified CEPs	Many applications (food, pharmaceuticals, and cosmetic)	Need for CEP production/extraction processes	Korhonen and Pihlanto, 2006; Sadat-Mekmene et al., 2011a; Gnasegaran et al., 2017
	No interferences with other compounds produced during fermentation	Poor CEP recovery from *Lactobacillus* cultures	
	Could increase peptide quantities	Optimal conditions for CEPs activity should be tested	
	Could be used in combination with commercial enzyme	An additional BAPs extraction/purification process is often needed	
		Scale-up limitations	


*BAPs, bio active peptides; CEP, cell envelope proteinase.*

**Table 2:** Applications of BAPs produced by *Lactobacillus* species depending on the production strategy.

Protein sources	Species	Activities	Reference
*Functionalization of food products*		

Cow milk	*Lb. helveticus*	ACE inhibitory	Quan et al., 2008; Wakai and Yanamoto, 2012
Cow milk	*Lb. helveticus*	Immunomodulation	Matar et al., 2001
Cow milk	*Lb. helveticus*	Bone mineralization	Narva et al., 2004a
Cow milk	*Lb. casei*	ACE inhibitory	Li et al., 2017
Cow milk	*Lb. casei*	Bone mineralization	Kim et al., 2009
Camel and cow milk	*Lb. plantarum, Lb. paraplantarum, Lb. kefiri, Lb. gasseri, Lb. paracasei*	Antioxidant	Soleymanzadeh et al., 2016
Pea	*Lb. plantarum*	ACE inhibitory	Jakubczyk et al., 2013
Soy milk	*Lb. plantarum*	ACE inhibitory, antioxidant	Singh and Vij, 2017

*Extraction or purification of peptides from fermented products*		

Cow milk or whey	*Lb. helveticus*	ACE inhibitory, antitumoral	LeBlanc et al., 2002; Ahtesh et al., 2016
Cow milk	*Lb. plantarum*	Antiinflammatory, antihemolytic, antioxidant, antimutagenic, and antimicrobial	Aguilar-Toalá et al., 2017
Cow milk	*Lb. acidophilus, Lb. helveticus, Lb. delbrueckii* subsp. *bulgaricus*	ACE inhibitory	Gandhi and Shah, 2014
Cow milk	*Lb. casei, Lb. rhamnosus*	ACE inhibitory, antioxidant	Solieri et al., 2015
Cow milk	*Lb. paracasei*	Hypertensive	Cheng and Pan, 2017
Cow milk	*Lb. casei*	ACE inhibitory	Han et al., 2012
Cow milk	*Lb. delbrueckii* subsp. *bulgaricus*	ACE inhibitory, immunomodulation	Kliche et al., 2017
Camel and cow milk	*Lb. plantarum, Lb. reuteri*	ACE inhibitory, α-glucosidase and α-amylase inhibitory, antioxidant	Ayyash et al., 2017
Bovine sodium caseinate	*Lb. acidophilus*	Antimicrobial	Hayes et al., 2006

*Utilization of partially purified CEPs for protein hydrolysis*		

Bovine β-casein	*Lb. acidophilus*	ACE inhibitory	Ren et al., 2015
Sodium caseinate (different sources)	*Lb. helveticus*	ACE inhibitory	Minervini et al., 2003
Cow milk	*Lb. delbrueckii* subsp. *lactis*	ACE inhibitory	Gnasegaran et al., 2017


*ACE, angiotensin converting enzyme; CEP, cell envelope proteinase.*

### Functionalization of Food Products for Improved Health Benefit

Fermentation with *Lactobacillus* bacteria has major applications in the food industry. In addition to modifying the organoleptic properties of the product, fermentation (i) improves its nutritional value, by increasing vitamin, amino acid, polysaccharide contents; (ii) decreases the immuno-reactivity of proteins by hydrolyzing allergenic epitopes, a phenomenon that was, for instance, studied on milk proteins hydrolyzed by *Lb. fermentum* and *Lb. delbrueckii* subsp. *bulgaricus* strains (El-Ghaish et al., 2011; Zheng et al., 2013; Pescuma et al., 2015); and (iii) brings extra benefits such as health-promoting properties, through generation of BAPs. Numerous BAPs have been found in fermented products, presenting different functional activities such as antihypertensive, immunomodulatory, hypocholesterolemic, antioxidative, antimicrobial, mineral binding, opioid, and bone mineralization activities (Marco et al., 2017). Many studies focused on ACE inhibitor peptides, probably due to the ease of use of *in vitro* anti-ACE assays. The well-known Val-Pro-Pro and Ile-Pro-Pro peptides are produced during milk fermentation by some *Lb. helveticus* strains. The resulting fermented milk showed antihypertensive activities in animal and human clinical studies, with a significant decrease in systolic blood pressure (Wakai and Yanamoto, 2012). An additional ACE inhibitory peptide sequence (Ala-Ile-Pro-Pro-Lys-Lys-Asn-Gln-Asp) was also identified in milk fermented by *Lb. helveticus* 130B4 strain isolated in Mongolia, demonstrating the potential of this species to release novel ACE inhibitor peptides (Quan et al., 2008). Moreover, other *Lactobacillus* species may also release new ACE inhibitory peptides, as reported for some *Lb. casei* strains (Li et al., 2017).

Other types of biological activities were also observed in fermented milk. Milk fermented by *Lb. helveticus* showed immunomodulating effect after administration to mice. This activity was not observed when mice were treated with milk fermented by a non-proteolytic variant of the same *Lb. helveticus* strain (Matar et al., 2001). Antioxidant activities were reported from cow and camel milk fermented by specific strains of *Lb. plantarum*, *Lb. paraplantarum*, *Lb. kefiri*, *Lb. gasseri*, and *Lb. paracasei* (Soleymanzadeh et al., 2016). Furthermore, a clinical study showed the possible impact of a milk fermented by *Lb. helveticus* on sleep efficiency in elderly subjects (Yamamura et al., 2009). Numerous animal or human clinical studies reported a positive impact of fermented milk on bone metabolism, probably because of the high calcium concentration and bioavailability in these products (Rizzoli and Biver, 2017). Indeed, fermented milk by *Lb. casei* 393 strain showed positive impact on bone metabolism in ovariectomized rats (Kim et al., 2009). A comparable effect was observed with milk fermented by *Lb. helveticus* strains on rats (Narva et al., 2004b) and even on calcium metabolism in postmenopausal women (Narva et al., 2004c). It was also demonstrated that a high intake of yogurt and dairy products was associated with antioxidative effects and a lowered risk of type 2 diabetes (Hove et al., 2015; Jin et al., 2016; Rutella et al., 2016; Fardet and Rock, 2017).

Because of their abilities to hydrolyze different proteins, *Lactobacillus* species are also used to produce fermented food from non-dairy sources. For instance, several *Lb. plantarum* strains were used for fermentation of pea seeds and soy milk, and the resulting products displayed ACE inhibition and antioxidative properties (Jakubczyk et al., 2013; Singh and Vij, 2017).

Fermentation by *Lactobacillus* species can, therefore, lead to the development of health-promoting food, allowing simultaneous functionalization and production. Actually, it must be noted that numerous metabolites are produced by *Lactobacillus* species during fermentation: not only BAPs, but also lactate, vitamins, or exopolysaccharides. All of these can contribute to the health- promoting effect (Fardet and Rock, 2017; Marco et al., 2017).

Another issue in BAPs research is that it is sometimes difficult to quantify one specific peptide in a whole hydrolysate or fermented food, and correlate its presence with the activity observed *in vitro* or *in vivo*. Actually, bioactivities of hydrolysates often result from the combined effects of several peptides.

Finally, a last issue to be kept in mind is that as a product designed for direct consumption, fermented food will be submitted to GID. Proteolytic enzymes (pepsin and pancreatic enzymes) present in the digestive tract will alter the peptide profile of a fermented food and change its activities *in vivo* (Daliri et al., 2017). The impact of GID on a fermented food must, therefore, be assessed to get an accurate prediction of the real *in vivo* activities of the food. In fact, this impact can be positive. GID is even, by itself, one of the main processes for BAP production from food proteins (Korhonen and Pihlanto, 2006). Usually, for ACE inhibitory peptides, GID increases the release of BAPs from fermented food, as proteins that were not hydrolyzed during the fermentation will be digested during GID. This was reported for fermented milk (Matar et al., 1996; Hernández-Ledesma et al., 2004; Jin et al., 2016). Furthermore, the Val-Pro-Pro and Ile-Pro-Pro sequences generated during *in vitro*/industrial fermentation are not altered by GID (Ohsawa et al., 2008). The impact of GID can be evaluated *in vitro* by treating the fermented product with gastric and pancreatic enzymes, and comparing the activity of the resulting product with the activity of a non-fermented sample.

### Extraction and Purification of Peptides From Fermented Media

The BAPs can be extracted and purified from fermentation broth. The resulting product will then be used for various applications (food, pharmaceutical, or cosmetic applications) as a functional or nutraceutical ingredient.

This approach involves production of a broth, from food products or byproducts, through fermentation with *Lactobacillus* species. The use of a non-proliferative medium, for instance, a casein suspension, is possible (Hebert et al., 2008). An advantage is that substrates other than food can be used as protein sources, especially industrial byproducts. For instance, *Lactobacillus* species can be used for the valorization of whey proteins from the cheese industry (Ahtesh et al., 2016).

Peptides generated during the fermentation are then isolated by a downstream process of extraction and/or purification. Methods for extraction and purification of peptides have already been reviewed (Korhonen and Pihlanto, 2006; Agyei et al., 2016). Selective precipitation is a fast, cost-effective, and easy to scale-up approach to separate peptides from proteins; trichloroacetic acid (TCA), ethanol, or ammonium sulfate (NH_4_)_2_SO_4_ are usually used as precipitating agents. For instance, TCA precipitation was used to obtain protein hydrolysates from *Lb. plantarum-*fermented milk (Aguilar-Toalá et al., 2017). The TCA was also used for the isolation of peptides from different milks fermented by *Lactobacillus* species (*Lb. helveticus, Lb. acidophilus, Lb. delbrueckii* susbp. *bulgaricus*, *Lb. casei*, and *Lb. rhamnosus*). The resulting hydrolysates displayed ACE inhibition or antioxidative activities (Gandhi and Shah, 2014; Solieri et al., 2015). Ethanol precipitation was used to purify fermentation broths of *Lb. paracasei*, which were used in animal studies for treatment of hypertension (Cheng and Pan, 2017). Approaches based on chemical precipitation, however, suffer from a major drawback: they require a cleaning step, such as chromatography or affinity columns, to remove the precipitating agent.

Another possibility is to take advantage of *Lactobacillus* species metabolism. Indeed, these bacteria produce lactic acid during fermentation, leading to a decrease of pH and, therefore, to the precipitation of proteins. Following fermentation, the water soluble extract (WSE) containing peptides can be readily separated by centrifugation (Ayyash et al., 2017). This approach is used preferentially because it does not require the use of chemical agents (Solanki et al., 2017; Taha et al., 2017).

Membrane ultra- and nanofiltration techniques can also be used for peptide extraction and purification (Coutte et al., 2017). Again, the addition of a chemical agent is not required. These procedures are easy to scale-up and can be combined with other processes. A membrane reactor can be used to allow a continuous process where protein hydrolysis and peptide separation will occur simultaneously (Wu et al., 2013; Hernández-Ledesma et al., 2014). Ultrafiltration was used for peptide separation from both fermented milk and casein suspensions. The cut-off threshold ranged from 3 to 14 kDa depending on the targeted peptides (Hayes et al., 2006; Han et al., 2012; Aguilar-Toalá et al., 2017).

Finally, chromatographic methods can be used to achieve peptide purification. These methods include size-exclusion chromatography and ion-exchange chromatography. They are highly selective, with a high-resolution separation. The main issue, especially for industrial applications, is the very high cost of these techniques. Furthermore, chromatography leads to peptide dilution and accumulation of solvent wastes (Agyei et al., 2016). For research applications such as peptidomics, chromatographic techniques are widely used. As an example, the purification and the preparation of antitumoral peptides were performed by gel filtration chromatography from a milk fermented by *Lb. helveticus* (LeBlanc et al., 2002). Due to its high resolution, this technique of purification is also particularly suitable for identification of the specific peptides involved in a given bioactivity. Peptide identification is indeed important to properly demonstrate that a given activity is associated with a specific BAP. Full identification and sequencing of active peptides are anyway necessary for pharmaceutical applications (Jin et al., 2016; Georgalaki et al., 2017; Giacometti and Buretić-Tomljanović, 2017).

Since the yield of BAP production using the proteolytic activity of *Lactobacillus* strains is relatively low compared with what is achieved using purified enzymes (Daliri et al., 2017), purification and extraction of BAPs will be useful as a tool for peptide enrichment in the final product. For example, a WSE of fermented milk by a strain of *Lb. delbrueckii* subsp. *bulgaricus*, presenting ACE inhibitory and immunomodulatory capacities, was lyophilized and used as a nutraceutical for yogurt (Kliche et al., 2017).

A major issue with this strategy is the cost of extraction and purification. It was estimated that about 70% of total peptide production costs are dedicated to downstream processes (Agyei et al., 2016). However, these steps are needed for research studies focusing on discovery and identification of BAPs. Moreover, it is also advisable to separate peptides from the fermentation broth to avoid interferences with other molecules (Daliri et al., 2017). Purification is also needed for specific nutraceutical or pharmaceutical applications where high-quality finished products are required.

### Utilization of Partially Purified CEPs for Protein Hydrolysis

The CEPs initiate protein breakdown in the extracellular media during fermentation. Fermentation, however, is not needed to achieve peptide production, the CEPs can be used as purified enzymes for *in vitro* hydrolysis. A controlled media for optimal enzymatic activity is requested, while optimal conditions for CEP activity can be different from those typically used for bacterial growth (Gnasegaran et al., 2017). Therefore, higher peptide concentrations can be reached in comparison with those obtained with fermentation approaches. Furthermore, the use of partially purified CEPs allows to generate peptides without production of other fermentation products, thus avoiding biological activity interferences (Daliri et al., 2017).

Obviously, a process of CEP purification is necessary before protein hydrolysis (Sadat-Mekmene et al., 2011a) as well as the assessment of optimal conditions for CEP activity (Ayyash et al., 2012). For example, different CEPs were isolated and purified or partially purified from *Lb. helveticus* strains. The resulting enzymes displayed a broad range of characteristics including a temperature optimum ranging from 40 to 50°C, a pH optimum ranging from 5.5 to 8, and, sometimes, a requirement for calcium ions as activators (Sadat-Mekmene et al., 2011a).

Depending on the species, CEPs are either anchored to the cell wall or released in the media. The CEPs from a *Lb. zeae* are secreted in the extracellular media, while several strains of *Lb. plantarum* seem to possess cell wall-bound CEPs (Vukotić, 2016). This feature determines which extraction strategy must be followed. Cell wall-bound CEPs will require proteinase extraction. For instance, CEP extraction from *Lb. helveticus* cell wall was carried out using proteinase treatment in Tris-EDTA buffer (Elfahri et al., 2014). The resulting crude CEP extract is then subjected to further enzyme purification process, like ion-exchange chromatography (Minervini et al., 2003). Bacterial culture conditions can be optimized to increase CEP production and extraction yields; this was done for *Lactobacillus* species such as *Lb. plantarum* (Kurniati et al., 2015), *Lb. delbrueckii* subsp. *bulgaricus* (Nour et al., 2014), or *Lb. delbrueckii* subsp. *lactis* (Agyei et al., 2012).

*In vitro* protein hydrolysis was carried out with extracted CEPs. The CEPs extracted from *Lb. acidophilus* JQ-1 were used for β-casein hydrolysis, leading to the release of ACE inhibitors (Ren et al., 2015). The CEPs from the *Lb. helveticus* PR4 strain were partially purified and used to hydrolyze six sodium caseinates from different milks (bovine, sheep, goat, pig, buffalo, or human milk). Among the generated hydrolysates, numerous ACE inhibitor peptides were identified as well as one antibacterial peptide derived from human β-casein (Minervini et al., 2003). Another study used the *Lb. delbrueckii* subsp. *lactis* ATCC7830 strain to produce CEPs (Agyei et al., 2012). This strain was also used for the development of an integrated process to produce ACE inhibitory peptides from milk. The process combined CEP production and milk hydrolysis followed by a downstream process of peptide purification. Although the process is expensive, the obtained results are promising for further industrial development (Gnasegaran et al., 2017).

Various strategies have also been tried to increase the yield of peptide production by CEPs. For instance, enzymatic immobilization (cross-linked CEP aggregates) was developed to increase activity and stability of CEPs (Agyei and He, 2015). The use of a combination of *Lactobacillus* CEPs and commercial enzyme is also another possible approach (Hafeez et al., 2014; Ahtesh et al., 2016).

## BAP Production by *Lactobacillus* Species: Limitations and Future Challenges

The use of *Lactobacillus* strains for BAP production is a strategy that still suffers from limitations.

First of all, CEPs are still poorly characterized enzymes. Their specificities are not clearly elucidated, and some of them probably remain to be discovered. There is a need for a comprehensive and comparative data analysis of fermentation potentials from different *Lactobacillus* strains, for example, through genomic comparison (Sun et al., 2015). To date, even a full comparative genomic study on different *Lb. helveticus* strains has yet not been performed (Stefanovic et al., 2017), although this species is considered as the most proteolytic among the *Lactobacillus* genus.

Peptidomics tools (chromatography, mass spectrometry, and bioinformatics) allow to analyze more rapidly and more accurately the heterogeneity of peptides produced through fermentation. Using computational methods (PCA, MLR, ANNs, and PLS-DA) (Dallas et al., 2016; Jin et al., 2016; Li et al., 2017), it is possible to visualize which protein regions are preferentially hydrolyzed by different lactobacilli and their CEPs. However, statistical tools are needed to deal with the large datasets generating by peptidomics as thousands of peptides can be identified in a single sample (Dupont, 2017). The collected data can then be assembled to create databases of fermented products (Giacometti and Buretić-Tomljanović, 2017), particularly, for milk proteins. which have been extensively studied.

The main limitation of *Lactobacillus* species use for BAP production is their low capacity to release peptides, compared with the activity of purified enzymes (Hernández-Ledesma et al., 2004; Daliri et al., 2017). This limitation could be addressed by designing improved fermentation media. For instance, a chemically defined medium was developed to increase the proteolytic activity of *Lb. delbrueckii* subsp. *bulgaricus* 761N strain (Nour et al., 2014).

Identification of improved strain variants or even metabolic engineering is another approach. Changes in metabolic pathways of different *Lactobacillus* strains were conducted successfully for different metabolisms like exopolysaccharides, lactic acid, vitamins, diacetyl, or even sorbitol (Papagianni, 2012; Stefanovic et al., 2017). However, to date, there are no studies on the metabolic engineering of the proteolytic system from lactobacilli. Research is needed to assess whether proteolytic capacities of promising *Lactobacillus* strains could be improved by CEP overexpression or coexpression of several CEPs by the same strain, which could increase the quantity and diversity of peptides produced upon fermentation.

These optimizations could be assisted by bioinformatics tools such as design of experiments (DOE) followed by response surface methodology (RSM) to improve BAPs release (Sánchez-Rivera et al., 2014; Nongonierma and FitzGerald, 2017a) or CEP production (Kurniati et al., 2015).

## Conclusion

This review highlights the great potential of *Lactobacillus* species to release numerous peptides (and potentially BAPs) during fermentation of proteins. Three different strategies were described to produce BAPs using *Lactobacillus* strains: (i) production and functionalization of dietary products by fermentation; (ii) extraction or purification of peptides from a fermented broth; and (iii) utilization of partially purified CEPs for protein hydrolysis. The choice between these strategies depends mostly on the targeted application.

In this review, a methodology was proposed for selecting efficient BAP-producing *Lactobacillus* strains (**Figure 2**). This method uses a combination of conventional (or empirical) approaches and *in silico* tools. By describing different strategies for BAP production and a method for strain selection, this work aims at delivering a general workflow for development of various BAP-containing products (nutraceuticals or functionalized foods) using lactobacilli.

Production of BAPs by *Lactobacillus* species is relatively inexpensive, compared to the use of purified enzymes. It also has a greater potential to generate new peptide sequences and, therefore, new BAPs with specific biological properties. However, there are still some challenges for further industrial exploitation of lactobacilli, such as the poor characterization of CEPs from different species and the low yields of peptide production by fermentation. Metabolic engineering of the proteolytic system of lactobacilli could overcome these challenges.

## Author Contributions

CR participated in all steps of preparation of this manuscript. BC, FC, CF, MF, DD, and PD participated in the editing of the manuscript and revised it critically.

## Conflict of Interest Statement

CR and MF are employed by the company VF Bioscience SAS, France. The remaining authors declare that the research was conducted in the absence of any commercial or financial relationships that could be construed as a potential conflict of interest.

## Abbreviations

ACE: angiotensin converting enzyme
BAPs: bio active peptides
CEP: cell envelope proteinase
DOE: design of experiment
DPP-IV: di peptidyl peptidase IV
EDTA: ethylene diamine tetra acetic acid
GID: gastro-intestinal digestion
LAB: lactic acid bacteria
MLR: multiple linear regression
PCA: principal component analysis
PLS-DA: partial least square discriminant analysis
QSAR: quantitative structural activity relationship
RSM: response surface methodology
TCA: tri chloro acetic acid
WSE: water soluble extract
